# Type II Toxin-Antitoxin Systems: Evolution and Revolutions

**DOI:** 10.1128/JB.00763-19

**Published:** 2020-03-11

**Authors:** Nathan Fraikin, Frédéric Goormaghtigh, Laurence Van Melderen

**Affiliations:** a Cellular and Molecular Microbiology (CM2), Faculté des Sciences, Université Libre de Bruxelles, Gosselies, Belgium; b Research Area Infection Biology, Biozentrum, University of Basel, Basel, Switzerland; McGovern Medical School

**Keywords:** persistence, proteolysis, stress responses, toxin-antitoxin, transcriptional regulation

## Abstract

Type II toxin-antitoxin (TA) systems are small genetic elements composed of a toxic protein and its cognate antitoxin protein, the latter counteracting the toxicity of the former. While TA systems were initially discovered on plasmids, functioning as addiction modules through a phenomenon called postsegregational killing, they were later shown to be massively present in bacterial chromosomes, often in association with mobile genetic elements. Extensive research has been conducted in recent decades to better understand the physiological roles of these chromosomally encoded modules and to characterize the conditions leading to their activation. The diversity of their proposed roles, ranging from genomic stabilization and abortive phage infection to stress modulation and antibiotic persistence, in conjunction with the poor understanding of TA system regulation, resulted in the generation of simplistic models, often refuted by contradictory results. This review provides an epistemological and critical retrospective on TA modules and highlights fundamental questions concerning their roles and regulations that still remain unanswered.

## Abstract (precis)

Type II toxin-antitoxin (TA) systems are small genetic elements composed of a toxic protein and its cognate antitoxin protein, the latter counteracting the toxicity of the former. While TA systems were initially discovered on plasmids, functioning as addiction modules through a phenomenon called postsegregational killing, they were later shown to be massively present in bacterial chromosomes, often in association with mobile genetic elements. Extensive research has been conducted in recent decades to better understand the physiological roles of these chromosomally encoded modules and to characterize the conditions leading to their activation.

## INTRODUCTION

**FIG 1:**
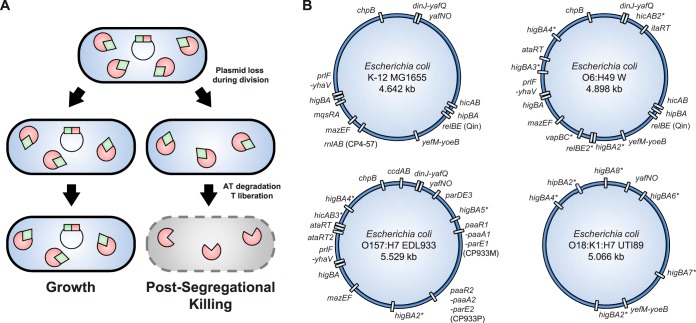
Type II TA systems, postsegregational killing and distribution. (A) Nonviable segregant or postsegregational killing model. TA genes, as well as proteins, are represented in red (toxins) and green (antitoxins). Rectangles denote TA genes encoded on a plasmid, and round shapes denote TA proteins produced from these genes. A TA-encoding plasmid can be lost during division in a way that one of the daughter cells does not inherit a plasmid copy. In these cells, TA proteins cannot be replenished due to the absence of TA genes. Since the antitoxin is degraded while its cognate toxin is stable, the free toxin concentration will increase, exert its activity, and, in time, induce cell death, therefore killing plasmid-free segregants. (B) Distribution of type II TA systems in various E. coli reference strains generated by TAfinder (23). Asterisks indicate systems that were not validated experimentally. Parentheses include name of the prophage a TA is encoded on when applicable. The strains are MG1655 (NCBI U00096.3), a common lab strain from phylogroup A; W (CP002967.1), a soil isolate from phylogroup B1; EDL933 (AE005174.2), an enterohemorrhagic pathogen from phylogroup E; and UTI89 (CP000243.1), a uropathogen from phylogroup B2. No TA systems are conserved within these four distantly related E. coli strains.

In 1983, while assigning functions to the open reading frames (ORFs) of the F plasmid, the Couturier group mapped a conditional lethal amber mutation to a novel ORF close to the replication origin of this plasmid and encoding a polypeptide named H (1). This mutation led to the induction of resident prophages in a *recA*-dependent manner (1). Interestingly, suppressor mutations could be mapped to the neighboring gene encoding a polypeptide named G (1). The authors concluded that the G protein is a potent inducer of the SOS response, whose action is negatively controlled by the H polypeptide (1). A simultaneously released publication from Ogura and Hiraga showed that these two genes were able to inhibit cell division when plasmid replication was hampered and the copy number decreased (2). These authors concluded that this locus promotes stable plasmid maintenance by coupling plasmid replication to host cell division (2). They named these two genes *ccdA* (H-encoding gene) and *ccdB* (G-encoding gene) for coupled cell division (2). In 1985, Jaffé, in collaboration with the group of Hiraga, showed that the *ccd* locus greatly reduced the viability of cells that failed to inherit a plasmid copy during division and proposed the “nonviable segregant model” (3, 4) (Fig. 1). The *ccd* locus was then defined as control of cell death (5). These genes constitute the first identified toxin-antitoxin (TA) pair, although this term was first used much later (6). Subsequent studies from the Couturier and Horiuchi groups concomitantly showed that the CcdB protein poisons DNA gyrase much like quinolone antibiotics, leading to the generation of double-strand breaks and induction of the SOS response (7–10). This provided the link with earlier observations showing that the *ccd* locus induces resident prophages and produces long nonviable plasmid-free filaments (1, 3). CcdA was shown to inhibit this DNA-damaging activity by directly interacting with CcdB (11, 12). CcdA was also shown to be unstable due to constitutive degradation by the Lon ATP-dependent protease, refining the earlier model proposed by Jaffé et al. (5, 13). Cells devoid of the plasmid would stop synthesizing the Ccd proteins. CcdA would then be degraded and not replenished, leading to the liberation of CcdB and killing of plasmid-free segregants (Fig. 1A) (3, 13). Analogous systems located on different plasmids and phages were described concurrently, i.e., *hok-sok* (*parB*) and *kis-kid* (*parD*) on plasmid R1, *pem* on plasmid R100 (which proved to be identical to *parD*), *stbDE* on plasmid R485, *parDE* on plasmid RK2, and *phd-doc* on bacteriophage P1 (14–19). The mechanism by which TA systems kill plasmid-free cells is known as “postsegregational killing” (PSK) (Fig. 1A), and TAs themselves were referenced to as addiction modules (14, 20). Over the years, additional TAs were identified on plasmids but also on chromosomes (21–24). They were divided into different classes depending on the nature and mode of action of the antitoxin, the toxin always being a protein (for reviews, see references 25 and 26). This minireview will focus on type II TA systems in which both components are proteins. This class of TAs appears to be the most abundant in bacterial genomes, being heavily represented in mobile genetic elements such as plasmids and phages but also in bacterial chromosomes (21–24). Since TA systems were described as stabilizers of mobile DNA, those encoded on chromosomes piqued the curiosity of the microbiology community and the study of plasmid TAs became neglected to the profit of chromosomally encoded ones (27).

### TA systems are abundant and part of the prokaryotic accessory genome.

With the advent of the genomic era, TA systems were being discovered in nearly every bacterial chromosome and, moreover, often in multiple copies. A first report was published in 2005 by the Gerdes lab in which a total of 126 bacterial and archaeal chromosomes were analyzed (21). At this time, only 5 and 38 type II TA systems were identified in Escherichia coli K-12 and Mycobacterium tuberculosis H37Rv, respectively (21). Several studies followed up on this initial report, mining hundreds of genomes and leading to the detection of thousands of TA systems in bacterial and archaeal genomes, as well as to the identification of novel families (22, 24). As the number of annotated TA systems has grown exponentially, it is now recognized that prokaryotic genomes are literally invaded by these systems. For example, 12 and 76 type II TA systems are currently detected in E. coli K-12 and M. tuberculosis H37Rv, respectively (23) (Fig. 1B).

One of these genomic studies also observed that obligate intracellular bacteria tend to harbor none or fewer TA pairs compared to related free-living species (21). Pandey and Gerdes argue that this observation supports a role for TA systems in stress responses, the rationale being the following: as obligate intracellular species thrive in stable niches and do not need to cope with changing environments, the selective pressure on TA systems is reduced, and these modules are eliminated through evolution (21). The authors also argued that, in addition to TA systems, obligate intracellular bacteria have also lost the genes encoding enzymes regulating the metabolism of (p)ppGpp, an alarmone involved in the stringent response, which is activated when cells are deprived of nutrients like amino acids (21). This observation thus supported an implication of TA systems in stress responses, as well as a functional link between these systems and (p)ppGpp (21). Intriguingly, the small genomes of intracellular bacteria consist of a stable chromosome in which the proportion of accessory genes is drastically reduced compared to free-living organisms. They are mostly devoid of mobile genetic elements such as plasmids, phages, transposons or integrative and conjugative elements, which are assumed to be lost during the reductive genomic processes that characterize the evolution of these intracellular species (28). We thus argue that this observation indicates that TAs are intimately linked to mobile DNA elements as proposed in (22, 24). For example, the *rnlAB* and *relBE* modules are located on the CP4-57 and Qin cryptic prophages of E. coli K-12, respectively (29, 30) (Fig. 1B). Enterohemorrhagic E. coli O157:H7 encodes two homologous three-component *paaR-paaA-parE* systems in cryptic prophages CP933M and CP933P (Fig. 1B) (31). Comparison of the TA distribution among the E. coli chromosomes shows that TA content is highly variable from one isolate to another, with few to no systems being universally conserved in a given species (32, 33) (Fig. 1B). These observations suggest that chromosomal TAs are acquired by horizontal gene transfer and establish independently or as part of genomic islands (32, 33). Other studies aiming at building an inventory of the TA repertoire encoded on various E. coli chromosomes showed that their locations are conserved and that some of these systems are degenerating (32, 34). This decay—reflected by deletion or accumulation of nonsense mutations—would indicate that TA systems are progressively lost during evolution. The reasons for decay might be multiple and will be discussed in light of their proposed functions below.

## PROPOSED FUNCTIONS: THE MANY FACES OF TA SYSTEMS

The detection of TA systems on chromosomes naturally led to the idea that these systems could have functions beyond plasmid stabilization. Here, we briefly describe the proposed functions of TA systems in E. coli, unless otherwise specified, and the recent tumultuous developments in that respect (for more general reviews on TA systems, see references 25 and 26).

### TA systems as stress response modules.

Early work on chromosomal TA systems showed that the activities of the RelE and MazF toxins in E. coli K-12 were triggered by amino acid starvation, leading to reduced translation during nutritional stress in a way that is independent of the (p)ppGpp alarmone (35, 36). This model was supported by the observation that amino acid starvation triggers mRNA cleavage in a *mazEF*- and *relBE*-dependent way (35, 36). Since RelE and MazE-induced toxicities are bacteriostatic and can be relieved by the *ssrA* tmRNA, bacteria could recover upon relief of the nutritional stress (36, 37). The authors proposed that in contrast to the stringent response, which inhibits translation indirectly, activation of TA systems would directly reduce translation and assist the stringent response in keeping energy consumption low under starvation conditions (35). In another series of studies, various stress conditions, such as nutritional stress, antibiotic exposure, oxidative stress, thymineless death, high temperature, and extracellular death factor (a linear signaling pentapeptide), were shown to mediate programmed cell death (PCD) solely through activation of the *mazEF* system in a (p)ppGpp-dependent way (38–43), in total disagreement with the data concerning *mazEF* involvement in nutritional stress management. Another series of studies claimed that the MazF RNase induces specific cleavages that can remodel cell physiology (44, 45). Transcriptome sequencing (RNA-seq) showed that MazF cleaves many messengers at or closely upstream of their translation initiation codon, thus generating leaderless mRNAs (44). This pool of leaderless transcripts was shown to be translated by a subpopulation of specialized ribosomes which were deprived of their anti-Shine-Dalgarno sequence by cleavage of the 23S rRNA by MazF (44). This was later shown to profoundly remodel the proteome of E. coli and to reflect a response induced by MazF when this system is activated under stress conditions (45). However, two independent studies failed to detect either leaderless transcripts or specialized ribosomes by RNA-seq (46, 47). Indeed, MazF was shown to cleave most messengers at multiple sites in their coding sequences as well as rRNA precursors, thus inhibiting ribosome biogenesis (46, 47). Reanalysis of previously published proteomic data using more powerful statistical analyses also failed to detect proteins upregulated in a MazF-dependent way during stress (48). Therefore, MazF was proposed to generally inhibit translation by cleaving transcripts indiscriminately and by cleaving rRNA precursors (46–49). The theory that TAs provide competitiveness under stress conditions was also put into question since deletion of five TA systems, including *relBE* and *mazEF*, did not cause any fitness deficiency under various stress conditions, such as nalidixic acid or rifampin exposure, amino acid starvation, nutritional downshift, low pH, or long-term stationary phase (50). Later on, the *mqsRA* system in E. coli was found to regulate a variety of biological processes such as biofilm formation and response to oxidative stress, notably by the capacity of the antitoxin MqsA to regulate expression of master regulators such as *csgD* or *rpoS* (51, 52). However, none of these phenotypes or regulations could be reproduced in an independent study (53). All of these observations call into question the implication of TA modules in various stress responses, whether through PCD, translation inhibition, or pleiotropic regulation.

Another pivotal role for TA systems consisted in the generation of persister cells, a subpopulation of supposedly dormant cells that is able to survive antimicrobial treatments. This hypothesis stemmed from the fact that missense mutations in the *hipBA* system induce a high proportion of persister cells and that other TA systems (*dinJ-yafQ*, *mazEF*, *mqsRA*, *relBE*, and *yefM-yoeB*) were highly expressed in populations enriched in persisters (54–56). A key study supporting this model showed that successive deletions of ten mRNase TA modules in E. coli K-12, which are among the most studied chromosomal TAs, lead to a proportional decrease in survival upon antimicrobial treatment (57). Complementary single-cell analyses based on fluorescent reporters concluded that antibiotic persister cells constitute a subpopulation in which the stringent response and TA systems are activated (58). This model was further refined by a study placing the *hipBA* module upstream and as a master regulator of the signaling cascade leading to persistence, proposing that stochastic activation of *hipBA* would lead to glutamate starvation since HipA inhibits glutamyl-tRNA charging (59). This would, in turn, activate the 10 TA-encoded mRNase toxins in a (p)ppGpp, polyphosphate and Lon-dependent manner, shutting down translation and leading to dormancy and antibiotic tolerance (58, 59). This model was, however, later refuted by multiple studies due to major experimental flaws (60–62). While these findings resolved the controversy surrounding the role of TA systems in antibiotic persistence in E. coli, a parallel story emerged in Salmonella enterica. Helaine et al. showed that, upon phagocytosis of S. enterica by macrophages, transient acidification and nutrient starvation would trigger activation of all 14 type II TAs, resulting in the generation of antibiotic persisters (63). An independent study was unable to observe reduced antibiotic persistence of TA-deleted S. enterica grown in medium mimicking conditions encountered in phagosomes such as low magnesium, amino-acid starvation or acid shock (64). A mutant deleted for three TA systems (*ecnB*, *shpAB*, and *phd-doc*) was also found to have no effect on virulence and persistence in a murine model (65). Further research will be required to clarify whether TA systems are involved in persistence into the host and whether they are involved in virulence, as is often claimed, although with very little mechanistic evidence (63, 66–68) or with experimental flaws, i.e., by ectopically overexpressing a toxin to study its effect on persistence (69–72; for a review, see reference 73).

As a conclusion, the lack of solid evidence to support the involvement of TA systems in the regulation of bacterial physiology, in spite of the attention accorded to these modules in the last 10 years, suggests that chromosomally encoded TA systems might provide other functions to their hosts.

### TA systems as selfish genes driving competition between replicons.

Alternative TA system functions, in accordance with their mobile and addictive nature, have been proposed. Similar to what is observed for plasmids, TAs were shown to stabilize large genomic elements such as superintegron arrays in chromosome II of Vibrio cholerae in the absence of selection, potentially providing a fitness advantage when coping with changing environments (74). Similarly, some TAs promote maintenance of genetic mobile elements such as cryptic prophages and conjugative transposons: for instance, *mosAT* stabilizes the SXT conjugative transposon of V. cholerae (75) and *paaR2-paaA2-parE2* is thought to stabilize the CP933P prophage of E. coli O157:H7 (31).

**FIG 2:**
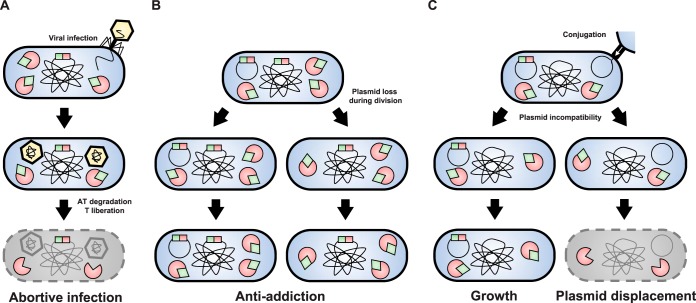
Roles of TA systems regarding mobile genetic elements. TA genes, as well as proteins, are represented in red (toxins) and green (antitoxins). (A) Protection against phages. Some TA systems have been shown to contribute to viral defense through a process known as “abortive infection.” Viral infection would lead to a molar excess of toxin over its cognate antitoxin by yet-unknown mechanisms, leading to the killing of infected cells, preventing phage replication and propagation. (B) Antiaddiction. Chromosomal homologs of plasmid-encoded TA systems can cross-neutralize their toxic activities. Therefore, failure to inherit a TA-encoding plasmid will not lead to postsegregational killing if a homologous TA system is encoded on the chromosome. (C) Plasmid displacement. Cells that acquire more than one plasmid from the same incompatibility group through conjugation will partition these plasmids in different daughter cells. If one of such plasmids encodes a TA system, cells that fail to inherit this plasmid will still contain TA proteins in its cytoplasm and will be killed by postsegregational killing.

Research published by Fineran and colleagues showed that type III and IV TA modules function as “abortive infection” systems, inducing cell death in cells infected by phages to prevent infections from spreading (Fig. 2A) (76–78). In E. coli, similar mechanisms could involve type II TAs as a deletion of *mazEF* leads to increased P1 infection loads (79). Interestingly, the T4 phage harbors the *dmd* gene, which encodes a promiscuous antitoxin for two type II toxins: RnlA from the CP4-57 cryptic prophage of E. coli K-12 and LsoA from the pOSAK1_02 plasmid carried by E. coli O157:H7 (80). The *rnlAB* and *lsoAB* systems strongly reduce the growth of a *dmd* mutant of T4, suggesting that these two systems protect their hosts against phage infection (80). The ability of T4 to circumvent these two systems through *dmd* also suggests that phages and their hosts are involved in a continuous and ever-evolving arms race in which TA systems might play a significant role.

Similarly, chromosomal TAs were proposed to protect cells from addiction by neutralizing plasmid-encoded homologous toxins (Fig. 2B). Specifically, the chromosomally encoded CcdA antitoxin of Erwinia chrysanthemi was shown to protect cells against the toxic effect of its F plasmid-encoded CcdB toxin homolog upon plasmid loss (81). Similarly, the *ataRT* system in the E. coli O157:H7 chromosome was shown to counteract toxicity of a plasmid-encoded homologous system, indicating that this system might play an antiaddictive role as well (82). Consequently, integration of TA systems from plasmids into chromosomes could be selected as a way to free a host from the burden of addiction (33, 83). The ability to neutralize addictive TAs could lead, in turn, to the selection of plasmid-encoded TAs that can circumvent neutralization by their chromosomal homologs, thus driving an arms race between the host and its accessory replicons and accounting for the diversity and multiplicity of TA systems, similarly to that proposed for restriction-modification (RM) systems (83, 84). As a result, chromosomal TA systems that would lose the capacity to counteract newly selected plasmid TA system will fall under neutral selection, albeit at a lower rate than other genes due to their addictive properties, explaining the decay previously observed for some of these systems (32, 34).

An interesting model suggested that TA systems could mediate competition between incompatible plasmids for the same host through PSK (Fig. 2C). TA systems allow a conjugative plasmid (TA^+^ plasmid) to outcompete a conjugative plasmid belonging to the same incompatibility group (identical replicon) but devoid of the TA system (TA^−^ plasmid). Cells that do not inherit a TA-encoding plasmid during this “conflict” will be subjected to PSK provided they do not carry a copy of this TA (Fig. 2C) (85). Therefore, TA-encoding plasmids can mediate the displacement of plasmids devoid of TA, ensuring both their vertical and horizontal inheritance through the same addiction mechanism (85). Interestingly, similar displacement mechanisms were observed for plasmids encoding RM systems. Like RM systems, TA modules are refractory to gene efflux at the expense of their hosts and were dubbed “selfish genes” (84–88). In fact, plasmid stabilization, displacement and antiaddiction might just be the consequence of the selfish properties of TA systems. Selfishness might allow chromosomal TA systems to subsist in their host without providing any function. Time and genetic drift may lead to the appearance of inactive toxin mutants, explaining why some of these systems are under negative selection and slowly decaying (32, 34).

## ACTIVATION OF TA SYSTEMS: FROM REGULATION TO PHENOTYPES

### Transcriptional regulation of TA systems.

**FIG 3:**
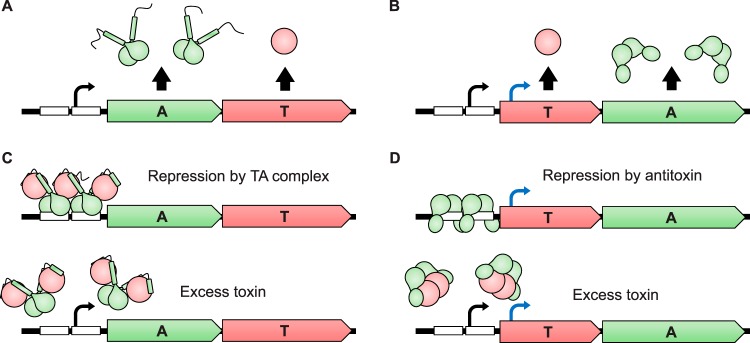
Transcriptional regulation of type II TA systems. TA genes, as well as proteins, are represented in red (toxins) and green (antitoxins). (A) Transcription of canonically organized TA systems. The whole operon (antitoxin-toxin) is transcribed by a single autoregulated promoter. A lower translational efficiency of toxins ensures a molar excess of antitoxin. (B) Transcription of reverse-organized TA systems. The whole operon is generally transcribed by a single autoregulated promoter (black arrow). A molar excess of antitoxin is ensured through its exclusive transcription by other promoters (blue arrow). (C) Conditional cooperativity. Unsaturated toxin-antitoxin complexes tightly bind their operators (white boxes) to repress transcription. A molar excess of toxin leads to the formation of saturated complexes that do not bind their operators, leading to derepression of the promoter, transcription of the operon, and *de novo* antitoxin synthesis. (D) Repression of reverse-organized systems. Excess antitoxin binds operators (white boxes) to repress transcription of the whole operon while the antitoxin gene is still transcribed. A molar excess of toxin displaces the antitoxin from its operators, leading to derepression of the autoregulated promoter (black arrow) and transcription of the whole operon.

Although multiple functions were proposed for TA systems as described above, TA expression and regulation have been given little attention despite their proposed importance in bacterial physiology. It is important to note that among the 12 type II TA systems characterized in E. coli, eight adopt the “canonical” genetic organization in which the antitoxin gene precedes the toxin gene (Fig. 3A), while the four others (*rnlAB*, *mqsRA*, *higBA*, and *hicAB*) are in “reverse” organization with the toxin gene being upstream of the antitoxin gene (89) (Fig. 3B). In both configurations, TA systems are transcribed as bicistronic operons by an autoregulated promoter (89) (Fig. 3A and B). In the case of “reverse” systems, additional promoters have been shown to play a role in antitoxin expression (Fig. 3B). For instance, the *rnlA* and *mqsA* antitoxin genes are transcribed from additional constitutive promoters located in the toxin coding sequence (53, 90). Another layer of complexity is found in the *hicAB* system: the *hicAB* autoregulated promoter produces a transcript truncated at the position of the *hicA* ribosome-binding site, thus only supporting synthesis of the HicB antitoxin (91). Another constitutive promoter upstream of the former transcribes a messenger supporting translation of the whole system (91).

TA system expression is tightly autoregulated. Antitoxins generally comprise a DNA-binding domain that binds inverted repeats located in the operon promoter to repress transcription of the system, with low affinity in most described cases (92–95). This affinity can be enhanced by forming a complex with its cognate toxin (92–95). Toxin binding also allows cooperative recruitment of additional antitoxins to remaining operators, strengthening the repression (96) (Fig. 3C). Exceeding a certain toxin/antitoxin ratio will induce the formation of saturated complexes that cannot bind their operators (96, 97) (Fig. 3C). Since antitoxins bind cooperatively to their operators but only under specific condition*s*, i.e., in a range of toxin/antitoxin ratios, this mechanism was dubbed “conditional cooperativity” (97). This mode of regulation was proposed to form a tight negative-feedback loop that buffers the system against fluctuations in transcriptional activity (98). Indeed, since antitoxins appear to be more efficiently translated than their cognate toxins (see below), derepression of a TA operon by an excess of toxin should favor antitoxin neosynthesis, thus allowing homeostatic maintenance of a low toxin/antitoxin ratio (98). In some cases, i.e., *mqsRA* and *hicAB*—systems in which the toxin gene precedes that of the antitoxin—toxins were shown to displace their cognate antitoxins from operator sequences upon binding and not act as corepressor as mentioned above (91, 99) (Fig. 3D). Antitoxins of reverse-organized systems from other species were also shown to lose affinity for their operators upon toxin binding, i.e., GraA from Pseudomonas putida and HigA from Proteus vulgaris (100, 101). Therefore, while conditional cooperativity is supposedly the prevalent mode of TA autoregulation, other modes of regulation might exist, especially for systems in “reverse” configuration as described above.

Various TA systems were also shown to be regulated by factors in *trans*. The *yafNO* module was shown to be regulated by the SOS response (102). This is due to this system being inserted immediately downstream of the *dinB* gene, which encodes DNA polymerase IV, a translesion synthesis DNA polymerase implicated in adaptive mutation (102). The *dinB* gene is itself repressed by LexA and constitutes an operon with *yafNO*, thus explaining why this system is under SOS control (102). However, deletion of *yafNO* has no effect on the induction of the SOS system, in adaptive mutation or survival to DNA-damaging fluoroquinolone antibiotics (61, 102). IHF and Fis, two proteins that participate in the topology of the nucleoid, bind upstream of the *hipBA* and *mazEF* promoters, respectively, and upregulate their transcriptional activities (103, 104). Crp-Sxy, a supposed regulator of competence, was also shown to stimulate transcription of the *hicAB* transcript (91). However, the role of these transcriptional regulators in the functionality of the TA systems they regulate is unknown.

### Posttranscriptional regulation of TA systems.

Ribosome profiling analysis performed with E. coli K-12 grown in synthetic medium suggests that all TA systems are expressed (89, 105). However, the translation efficiency of toxins appears to be lower relative to antitoxins. For example, the RelB antitoxin is translated six times more efficiently than its cognate toxin (105). This should allow a molar excess of antitoxin to ensure that the entire pool of cellular toxin is neutralized. Translational coupling and transcript cleavage at the level of the toxin sequence were shown to maintain a molar excess of antitoxin in the *parD* system (106). Analysis of RNA-seq data detected truncated transcripts of the *dinJ-yafQ* and *yafNO* systems in the toxin reading frame, suggesting that these two systems maintain higher antitoxin levels through mechanisms similar to *parD* (89).

### Posttranslational regulation of TA systems.

Previous studies on plasmid-encoded TA systems have determined that antitoxins were proteolytically unstable. For example, CcdA is actively and constitutively degraded by the Lon protease (107). Other antitoxins, like Kis, from the *parD* system or Phd were shown to be degraded in a similar way by the ClpAP and ClpXP proteases, respectively (108, 109). Therefore, constitutive proteolysis of plasmid-encoded antitoxins appears as a rule since it supposedly allows the liberation of toxin molecules in plasmid-free segregants.

As mentioned above, *relBE* and *mazEF* were first thought to be involved in nutritional stress responses (35, 36). Amino acid starvation induced by serine hydroxamate [SHX; which inhibits serine incorporation into proteins and triggers (p)ppGpp synthesis] was shown to activate the degradation of the RelB and MazE antitoxins independently of (p)ppGpp (35, 36). The authors of those studies proposed that amino acid starvation would trigger proteolysis of these two antitoxins. However, since SHX by itself is a translation inhibitor (35, 110), it is likely that the observed degradation of RelB and MazE under such treatment is simply a secondary effect of antitoxin instability and neosynthesis inhibition. Indeed, similar degradation kinetics were measured for RelB in the presence of chloramphenicol, a potent translation-inhibiting antibiotic which does not promote (p)ppGpp synthesis (35). A few years later, in the context of bacterial persistence, the same authors came up with a model proposing that (p)ppGpp (in contradiction to what is mentioned just above) induces antitoxin degradation through activation of the Lon protease by polyphosphate accumulation (58). This regulation scheme has been, however, contradicted by many studies. Antitoxin degradation under SHX treatment was shown to be (p)ppGpp independent on multiple occasions (35, 36, 62). In addition, it was shown than Lon is inhibited by polyphosphate *in vitro* and that (p)ppGpp does not affect polyphosphate synthesis (111, 112).

To further study antitoxin degradation, the overproduction of Lon was used as a tool to destabilize antitoxins, with the aim of releasing toxins and studying subsequent phenotypes. Under these overexpression conditions, growth and translation were inhibited and mRNA cleavage was detected as well (113). Deleting the five TA systems known at the time (*yefM-yoeB*, *relBE*, *mazEF*, *chpB*, and *dinJ-yafQ*) only partially rescued growth and translation inhibition but completely eliminated mRNA cleavage (113). Interestingly, rescue was solely provided by deletion of the *yefM-yoeB* system, while deletion of the four other TAs had no effect (113). This suggests that, under these conditions, only *yefM-yoeB* is activated through Lon-dependent proteolysis of the YefM antitoxin. However, while massive overproduction of Lon can bring some insight into its effects on the *yefM-yoeB* system, this does not constitute a physiologically relevant condition. A subsequent study from Janssen et al. demonstrated that *yefM-yoeB*-dependent mRNA cleavage is induced in cells grown at 42°C in a *lon*-dependent manner and in a background preventing ribosome rescue from YoeB (*ssrA* mutant) as well as stalled mRNA degradation (*rnb* mutant) (114). However, the authors found that *yefM-yoeB*-induced cleavage at 42°C was not correlated with growth inhibition and that *yefM-yoeB* did not give any fitness advantage or disadvantage when cells were grown at this temperature (114). Finally, another study showed that the MqsA antitoxin is rapidly degraded by Lon under oxidative stress (115), which was not reproduced by a subsequent study (53). To conclude, antitoxin proteolysis under stress conditions, leading to TA system activation, is far from being a general rule, and further research is needed to better understand the conditions leading to TA systems induction and phenotypes, probably on a case-by-case basis.

## WHERE WE STAND, WHERE WE GO: QUESTIONS FOR THE FUTURE BASED ON LESSONS FROM THE PAST

First reports on chromosomally encoded TA systems date back to the late 1990s—about 10 years after their discovery on plasmids—when a homolog of R100 *pem* system (*mazEF*) and a new TA (*relBE*) were identified in the chromosome of E. coli K-12 laboratory strains (29, 38, 116). Mutants of the *relB* gene were actually first isolated in the late 1970s and conferred the so-called “delayed-relaxed” phenotype in which stable RNA synthesis continues for 10 min after amino acid starvation, accompanied by a reduction of (p)ppGpp concentration and synthesis of a translation inhibitor, most likely RelE (117). These mutants were phenotypically and genetically different from the classical “relaxed mutants” mapped in the *relA* gene, a finding which led to the suggestion that different loci could be involved in the stringent response (117). It was later shown that one of the *relB* alleles—*relB101*—encodes a destabilized RelB antitoxin that is more susceptible to Lon-mediated proteolysis than the wild-type RelB and that the “delayed-relaxed phenotype” is dependent on the RelE toxin (118). The *mazE* gene was first identified as an ORF of unknown function (*ma-ze* meaning “what is it” in Hebrew) located downstream of the *relA* gene (119). It was further shown that the *mazEF* locus is comprised in an operon together with *relA* (the latter having its own promoter) (38, 119).

These early observations naturally led to hypotheses linking TA systems to the stringent response that were compelling to investigate. Subsequent works proposed that TAs were linked to nutritional stress responses but with contradicting regulation and outcomes. In the case of *mazEF*, (p)ppGpp is thought to repress transcription of the operon, leading to the decay in *mazEF* mRNAs and degradation of MazE (38). Cells in which *mazEF* is “activated” would undergo programmed cell death due to MazF-dependent translation inhibition; cells in which *mazEF* is not activated would become necrophagic, surviving by feeding on their dead kin (41). In the case of *relBE* (also shown for *mazEF*, which directly contradicts the data mentioned just above), (p)ppGpp was proposed to increase transcription of the *relBE* and *mazEF* operons due to degradation of RelB and MazE (35, 36). In this model, cells in which *relBE* is “activated” would be metabolically dormant due to RelE-dependent translation inhibition and protected against stress; cells in which *relBE* is not activated would not survive (35). For both systems, the roles further expanded with time with, for example, MazEF becoming involved in an impressive number of stress responses—evolving into a “universal” stress-managing module capable of sensing many types of environmental changes and adjusting cell physiology accordingly, by generating specialized ribosomes and leaderless transcripts (38–45). The idea of a “stress regulon” controlled by TAs was further expanded with a TA system in M. tuberculosis that would hypothetically rewire the proteome toward the expression of stress proteins (120). Ultimately, *relBE*, as well as *mazEF* and other TA systems, became pivotal effectors of antibiotic persistence in a unified model in which all these systems would act synergistically and redundantly (58).

With time and in a common effort from independent groups, these models were questioned or disproved by different studies, shedding light on conceptual issues—notably, the absolute necessity that TA systems have to carry out important functions in cellular physiology since they are so widespread in bacterial chromosomes—as well as major experimental flaws like strain genotype issues, fluorescent reporter issues, and questionable data analysis (46, 47, 53, 60, 61). We thus propose to go back to the basics, ask crucial questions that still remain unanswered, and provide suggestions which could, hopefully, improve our understanding of TA systems.

### When and how TA systems are activated?

Many studies claim that TA systems are activated under specific conditions by showing transcriptional upregulation of these systems under such conditions (55, 56, 63, 67, 70, 121, 122). As mentioned above, translation of antitoxins is more efficient than their cognate toxin. It is thus likely that transcriptional derepression and upregulation of TA systems allows the synthesis of supplemental antitoxin. Therefore, observing transcriptional upregulation of a TA system does not imply that free toxin is present in molar excess and is able to exert its activity. Another interesting observation is that toxins interact tightly with their cognate antitoxins, with dissociation constants in the nanomolar range (99, 123). Therefore, one could wonder whether toxin dissociation from its antitoxin is spontaneous and supported by antitoxin degradation or, rather, an active process involving factors in *trans* like chaperones.

### What are the consequences of TA activation?

Should TA systems be activated, what would be the free toxin concentrations required to inhibit growth or induce cell death? As mentioned earlier, toxin activities were reported in physiologically relevant conditions but failed to induce any observable phenotypes (35, 36, 50, 114). Therefore, are there conditions in which free toxins are present at sufficient concentrations to have a discernible effect on cell physiology? Do TA genes necessarily need to be lost to see the negative effects of toxin liberation? If there are conditions in which the free toxin concentration is high enough to have an effect on cell physiology, would these toxins transiently inhibit growth or irreversibly affect cells? Can the effects of toxin activities be reversed? Can toxins be neutralized and cell growth resumed? These questions have been scarcely addressed and studied mainly by finding suppressors of toxin activity. For example, the *ssrA* tmRNA was shown to strongly curtail toxicity of translation-dependent mRNase toxins such as RelE or MazF, opening the possibility that the effects of these toxins can be reversed by *trans* translation (36, 37). In another study, Cheverton et al. showed that the toxicity of TacT, a tRNA-acetyltransferase toxin from Salmonella enterica, could be suppressed by the overproduction of peptidyl-tRNA hydrolase (Pth), suggesting that TacT-dependent growth inhibition and dormancy can be rescued (124). Interestingly, overproduction of Lon greatly reduced viability of E. coli, in a way that is dependent on the *yefM-yoeB* system, as mentioned above (113). Viability was not rescued upon plating on inducer-free medium. Therefore, the activation of *yefM-yoeB* under these conditions is partially responsible for this loss of viability, suggesting that activation of this system irreversibly kills cells (113). Since most toxins broadly inhibit translation and probably block *de novo* synthesis of their cognate antitoxins, how would cells be able to produce antitoxins after the toxins have reached growth-inhibiting concentrations? To date, the study of the reversibility of TA activation is challenging simply due to the lack of physiologically relevant conditions that activate TA systems and produce discernible phenotypes.

### Why are TA systems so ubiquitous, highly dynamic, and mobile?

Since TA systems are part of the accessory genome, studying their origin and evolution may provide a way to understand their functions. As Fiedoruk and colleagues attempted to characterize the distribution and dynamics of TA systems in various E. coli strains, it was striking to see how heterogeneously these systems are distributed, with close to none of the 84 characterized strains having the same set of TA modules (32) (Fig. 1B). Thus, one can wonder how genes so erratically distributed and so impervious to fixation can be pivotal in essential processes such as stress responses. Thus, due to their heterogeneous distribution, it is likely that functions of TA systems are intimately linked to their mobile nature. Others have tried to assess functions of TA systems based on their locations, with the above-mentioned examples of *mazEF* and *yafNO* being part of larger operons (*relA-mazEFG* and *dinB-yafNOP*, respectively) but having no functional link with these other cistrons (50, 102, 119). It is, however, worth noting that in the many strains where these systems are absent, e.g., UTI89 (Fig. 1B), *relA-mazG* and *dinB-yafP* form undisturbed bicistronic operons (21, 32). It is thus likely that these operons are perfectly functional without TA pairs and that insertion of these systems in their intercistronic region occurred independently of their biological functions. In fact, TA insertion loci are quite plastic and can accommodate various mobile genetic elements. For example, a *parDE* system can be found in the *relA-mazG* intergenic region in *Salmonella* spp., while REP sequences can be found in the *folA-apaH* intergenic region that contains the *ccd* system in E. coli O157:H7, suggesting that TA insertion does not happen at random locations (21, 34). However, the mechanisms by which TA systems move between hosts and integrate into genomes remain unelucidated to this day.

### What are the functions of TA systems?

We would also like to address a few more fundamental concepts in the study of TA systems. First, it is widely acknowledged that point mutations in TA systems can be selected during various genetic screens, when isolating mutants highly tolerant to antibiotics or deficient for the stringent response, for example (54, 117, 125). While this surely shows that TA systems are highly plastic and that phenotypes can be selected in a laboratory context with stringent conditions, it does not demonstrate a function for the wild-type variants of these systems. Another concerning approach is the use of toxin overproduction to study the functions of TA systems. While it may be amenable to find the target of this toxin, it is a questionable approach to study its implication in biological processes. Many studies ectopically overexpressed toxins and observed a phenotype, i.e., increased antimicrobial tolerance, thus claiming that TA systems are implicated in these processes; yet deleting these systems would not induce the opposite phenotype (69–72). However, artificially providing a molar excess of toxin disregards the fact that antitoxins are always produced in excess and that TA systems have to be activated to produce the phenotype observed under overproduction conditions. Therefore, instead of studying phenotype using ectopic toxin overexpression, one should determine whether toxin activity (e.g., RNA cleavage) and TA-dependent phenotypes (using properly designed deletion mutants of the whole module) can be detected in conditions of interest. Moreover, with the development of single-cell methods, observing TA activation with proper fluorescent reporters within individual cells should provide valuable insights about regulations at different levels.

To conclude on a positive note, many questionable studies and other controversies have set the TA field back to the questions it looked to address ten years ago (“TA systems, why so many, what for?”) (126). However, this step back is an opportunity to make two steps forward and to make new discoveries on TA systems with a clean slate and without the preconceived ideas that plagued the field for many years. Provided that TA systems are studied with a critical eye, we believe that the future holds great promises for these small but ubiquitous modules.
